# Toward Hand Pattern Recognition in Assistive and Rehabilitation Robotics Using EMG and Kinematics

**DOI:** 10.3389/fnbot.2021.659876

**Published:** 2021-05-13

**Authors:** Hui Zhou, Qianqian Zhang, Mengjun Zhang, Sameer Shahnewaz, Shaocong Wei, Jingzhi Ruan, Xinyan Zhang, Lingling Zhang

**Affiliations:** ^1^School of Automation, Nanjing University of Science and Technology, Nanjing, China; ^2^Affiliated Nanjing Brain Hospital, Nanjing Medical University, Nanjing, China

**Keywords:** EMG, kinematics, hand pattern recognition, sensor fusion, machine learning

## Abstract

Wearable hand robots are becoming an attractive means in the facilitating of assistance with daily living and hand rehabilitation exercises for patients after stroke. Pattern recognition is a crucial step toward the development of wearable hand robots. Electromyography (EMG) is a commonly used biological signal for hand pattern recognition. However, the EMG based pattern recognition performance in assistive and rehabilitation robotics post stroke remains unsatisfactory. Moreover, low cost kinematic sensors such as Leap Motion is recently used for pattern recognition in various applications. This study proposes feature fusion and decision fusion method that combines EMG features and kinematic features for hand pattern recognition toward application in upper limb assistive and rehabilitation robotics. Ten normal subjects and five post stroke patients participating in the experiments were tested with eight hand patterns of daily activities while EMG and kinematics were recorded simultaneously. Results showed that average hand pattern recognition accuracy for post stroke patients was 83% for EMG features only, 84.71% for kinematic features only, 96.43% for feature fusion of EMG and kinematics, 91.18% for decision fusion of EMG and kinematics. The feature fusion and decision fusion was robust as three different levels of noise was given to the classifiers resulting in small decrease of classification accuracy. Different channel combination comparisons showed the fusion classifiers would be robust despite failure of specific EMG channels which means that the system has promising potential in the field of assistive and rehabilitation robotics. Future work will be conducted with real-time pattern classification on stroke survivors.

## Introduction

Stroke is currently the major cause of disability worldwide and more than 17 million people are estimated to suffer from stroke globally each year (Feigin et al., 2014). 80% acute stroke patients have upper limb motor impairment, and 50% of such post-stroke patients face reduced arm function problems even after 4 years (Bernhardt and Mehrholz, 2019). They experienced loss of sensation, capability, movement, and coordination leading to difficulties surrounding activities of daily living (ADL). Rehabilitation and assistive robotics represent promising treatment methods for post-stroke patients' upper limb recovery and further assist of ADL (Mehrholz et al., 2018). Since hand function recovery is one of the most important and challenging aspects post stroke, robot based neurorehabilitation and assisting of ADL is a research area of great social and clinical significance.

Electromyogram (EMG) has been extensively used in driving the rehabilitation and assistive robotics for stroke. The extracted information from EMG can be used as a trigger (Dipietro et al., 2005) or as a proportional control strategy in hand rehabilitation robotics (Lenzi et al., 2011; Song et al., 2013). Furthermore, current rehabilitation and ADL assistive devices only use simple extension-flexion mode, but more tasks such as hand opening, grip, lateral pinch, and fist etc. are of imperative need in stroke survivors as well. However, the hand pattern recognition of ADL tasks remain challenging (Lu et al., 2019). Some studies reported the classification results of ADL tasks with very mixed results. The test results from normal subjects in those experiments were promising (Chen et al., 2017; Castiblanco et al., 2020) but for some stroke subjects the accuracy was subpar at best (Lee et al., 2011; Geng et al., 2013; Lu et al., 2019). Hence, more research is needed to develop a practical and effective pattern recognition paradigm that can be used in the rehabilitation and assistive robotics of post stroke patients.

Motion capture technology has been used in many rehabilitation robots to record kinematics data during the task and assess the recovery process post stroke (Rose et al., 2018; Vermillion et al., 2019). Currently, the commonly used motion capture equipment are marker based multicamera motion capture system, such as Vicon and Motion Analysis, etc. However, these devices are expensive and the establishment of markers on the subjects are time consuming. On the contrary, some low cost and non-marker based motion capture technologies have been developed recently, such as Leap motion (Leap Motion Inc., San Francisco, CA, USA) and Kinect (Microsoft, USA). Compared with Kinect, the Leap Motion controller can provide more precise information of the hand and finger movements (Nizamis et al., 2018). Furthermore, Leap motion is widely used as an effective human computer interaction method that can recognize patterns with high accuracy (Lu et al., 2016; Mantecón et al., 2019; Nogales and Benalcazar, 2019; Li et al., 2020). Besides, some Leap motion based hand function assessment system have been developed to evaluate the recovery progress of post stroke patients (Cohen, 2020). Leap motion is fascinating in the area of rehabilitation since it can record hand kinematics data with ease of use, low cost and acceptable precision (Bachmann et al., 2018).

EMG and kinematic recording devices have been jointly used in the state of the art hand rehabilitation robotics for the generation of subject specific task kinematics and assessment of recovery progress post stroke (Rose et al., 2018; Vermillion et al., 2019). For applications in hand rehabilitation and ADL assisting robots, the joint use of EMG and Leap motion controller is an affordable method to obtain muscle activation and kinematics simultaneously from subjects. To our great knowledge, few papers have been reported about the use of EMG and Leap motion sensors together in the area of rehabilitation robotics. In a recent publication, EMG signals and joint trajectories were simultaneously recorded by MYO armband and Leap motion controller (Arteaga et al., 2020). In their work, EMG was used to classify five patterns and three different activation levels, while Leap motion was used to calculate joint trajectories. Besides, it was reported that EMG and Leap motion were used together to recognize hand pattern of six ADL tasks (Ricardez et al., 2018). Integrated myoelectric potential from eight channels were extracted and fused with joint angles to achieve an average classification of 87.3%. It is desirable to develop pattern recognition system which is robust to EMG channel failure and noises. The objective of this study is to develop a pattern recognition paradigm with EMG and kinematics for future use in rehabilitation and assistive robotics with high accuracy and ease use. The contribution of the presented paper will be:

1. Design and implementation of feature fusion and decision fusion of EMG and Kinematics based hand pattern recognition system of daily activities based on EMG and kinematics for post stroke patients.
2. Checking robustness of feature fusion and decision fusion classifiers by adding different levels of noise and failure of EMG channels.

In the following sessions, the methods and results of hand pattern recognition using feature fusion and decision fusion of EMG and kinematics are described and discussed. In session II, the recruitment of subjects, the design of daily activities and experimental protocol, experiment instrument and proposed classification framework are briefly explained. In session III, the hand pattern recognition results are compared between EMG, kinematics, feature fusion and decision fusion of EMG and kinematics. Furthermore, classification results of different EMG channel combination and fusion with kinematics are considered. A discussion is provided in session IV followed by conclusion.

## Methods

### Subjects

Ten healthy normal subjects (six male and four females, 20–24 years old) and five post stroke patients (three male and two female, 50–81 years old) participated in the experiment and normal subjects were students of Nanjing University of Science and Technology. All normal subjects had no hand movement disorders and were familiar with patterns and data collection procedures before the start of the experiment. Three post stroke patients' right side was affected and two patients' left side was affected after stroke. Hand corresponding to the affected side of post stroke patients were chosen for pattern recognition procedure. Impairment severity range of the post stroke patients were covered with score of FMA (Fugl-Meyer Assessment Upper Extremity) and FMA-C (Hand part of FMA) (Lu et al., 2019). Detailed information of the post stroke patients are given in Table 1. All subjects were informed about the purpose and experimental procedure of the study. The recruitment of subjects and the experimental protocols were approved by the Ethics Committee for Human Research, Nanjing Brain Hospital Affiliated to Nanjing Medical University.

**Table 1:** Details of five post stroke patients.

**Subject**	**Age**	**Gender**	**Months since onset**	**Affected side**	**FMA**	**FMA-C**
S1	76	Female	1	Left	44	14
S2	50	Male	2	Right	43	12
S3	58	Male	1	Right	64	14
S4	54	Male	4	Right	64	13
S5	81	Female	1.5	Left	63	13

*FMA, Fugl-Meyer Assessment Upper Extremity, a score between 0 (no function) and 66 (intact); FMA-C, Part C (Hand) of FMA, a score between 0 (no function) and 14 (intact)*.

### Experiment Protocol

**Figure 1:**
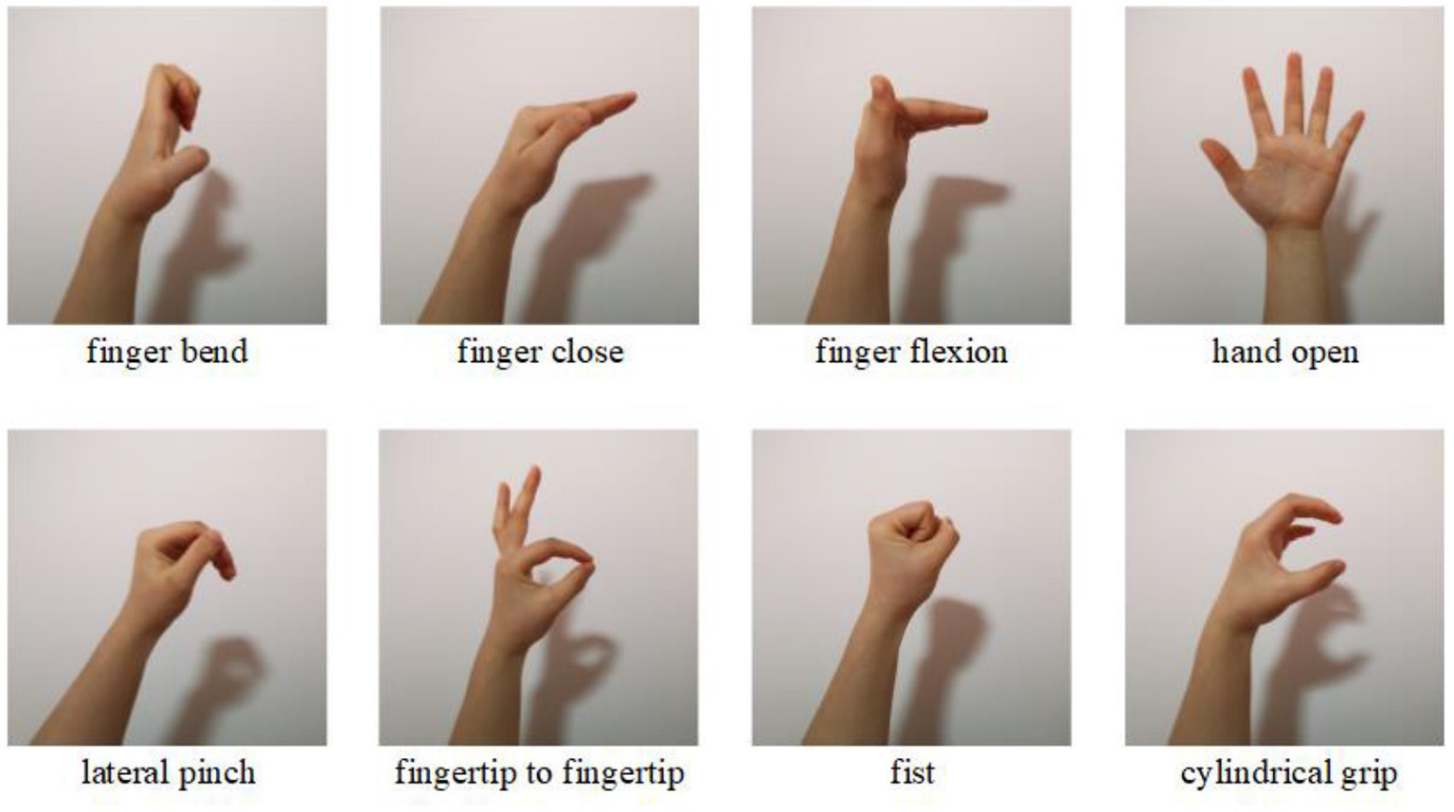
The eight different hand patterns tested in the experiment.

In the experiment, all the subjects were asked to sit on a chair and placed their hands loosely on the table surface. The subject were instructed to perform eight static hand patterns sequentially with their left hand for normal subjects and affected side hand for post stroke patients (finger bend, finger close, finger flexion, etc., see Figure 1). Each pattern was held for 5 s, followed by 10 s of rest to prevent muscle fatigue. The process was repeated 10 times for normal subjects and five times for post stroke patients.

### Instrumentation and Data Acquisition

**Figure 2:**
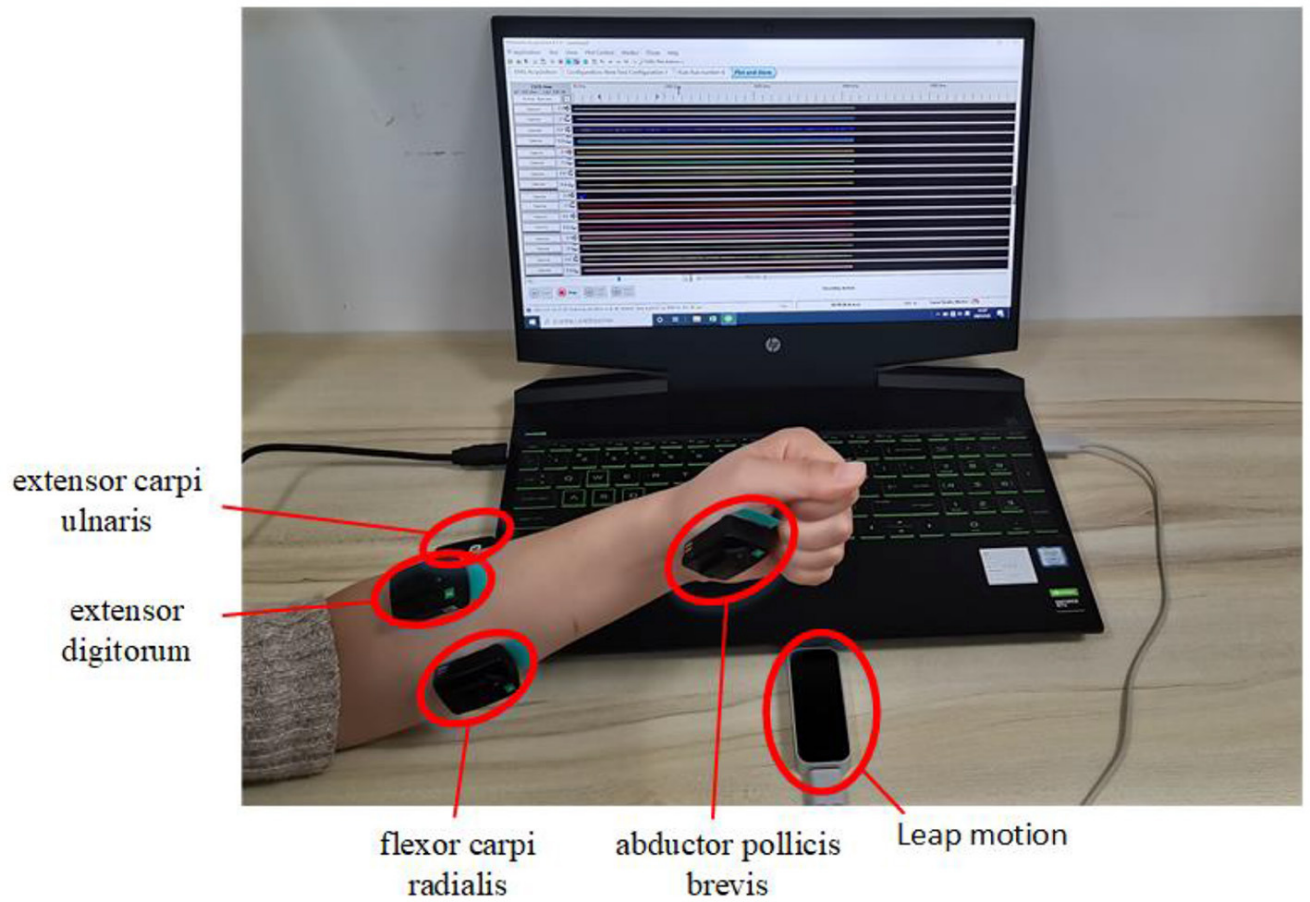
Demonstration of the experimental setup of EMG and kinematic sensors. EMG signals are recorded from muscles of abductor pollicis brevis, flexor carpi radialis, extensor digitorum, and extensor carpi ulnaris of the left hand and kinematics data are recorded from Leap motion sensor.

In the experiment signals from sensors of EMG and Leap motion were recorded at the same time. EMG signal were measured using the Trigno wireless EMG system (Delsys Inc., Natick, MA, USA) at sampling frequency of 2000 Hz (He et al., 2015). In the study, the muscles of abductor pollicis brevis, flexor carpi radialis, extensor digitorum and extensor carpi ulnaris of the left hand were chosen as the sites for myoelectrical recording, see Figure 2. For each subject, the skin of the recording muscles were cleaned with 75% alcohol prior to the start of the experiment, and then the EMG sensors were attached to the muscles with medical grade double sided adhesive tape. Additionally, a bandage (Kindmax Inc., Irvine, CA, USA) was used to fix the sensor to the upper limb to minimize EMG sensor movement and vibration during the tasks. Besides, an infrared motion sensor (Leap Motion Inc., San Francisco, California, USA) was also used to record hand kinematics at sampling rate of 20 Hz. In the study, the subjects were asked to place their left hand directly above the Leap motion controller, ~30–50 cm, see Figure 2. In addition, the participants were required to place their palm of left side (for normal subjects) or palm of affected side (for post stroke patients) facing the device.

### Feature Extraction

#### EMG Feature Extraction

The recorded EMG data were preprocessed via a second-order Butterworth band pass filter of 20–500 Hz. EMG signals of middle 3s were used in the analysis. A 250-ms-lengths sliding window with an overlap of 50 ms was used to segment the raw signal and features were extracted within the window. The following time domain features were used: mean absolute value (MAV), ZC (zero crossing), slope sign changes (SSC), difference absolute mean value (DAMV), and variance (VAR), (Englehart and Hudgins, 2003).

1) MAV

MAV detects the muscle contraction level. MAV is defined as the sum of the absolute value of the EMG signal, a moving time window of N samples is used to calculate it with the following algorithm:

$$\begin{array}{l} MAV=\frac{\text{1}}{N}\sum_{\text{i}=\text{1}}^{N}|x_{i}| \end{array}$$

2) Zero Crossings

Zero crossings refers to calculating the number of times the signal waveform passes through the zero point so that it can estimate the frequency domain characteristics.

3) Slope Sign Changes

This parameter calculates the number of sign changes of the signal slope. Similarly, it needs a threshold to reduce the interference caused by noise to the slope sign change.

4) DAMV

DAMV reflects the vibration characteristics of the EMG signal. It is calculated as follows:

$$\begin{array}{l} DAMV=\frac{1}{N}\sum_{i=1}^{N-1}|x_{i+1}-x_{i}| \end{array}$$

5) VAR

VAR is a measure of the power of the EMG signal. It can be calculated as:

$$\begin{array}{l} VAR=\frac{1}{N-1}\overset{N}{\underset{i=1}{\sum }}{x_{i}}^{2}. \end{array}$$

#### Leap Motion Feature Extraction

**Figure 3:**
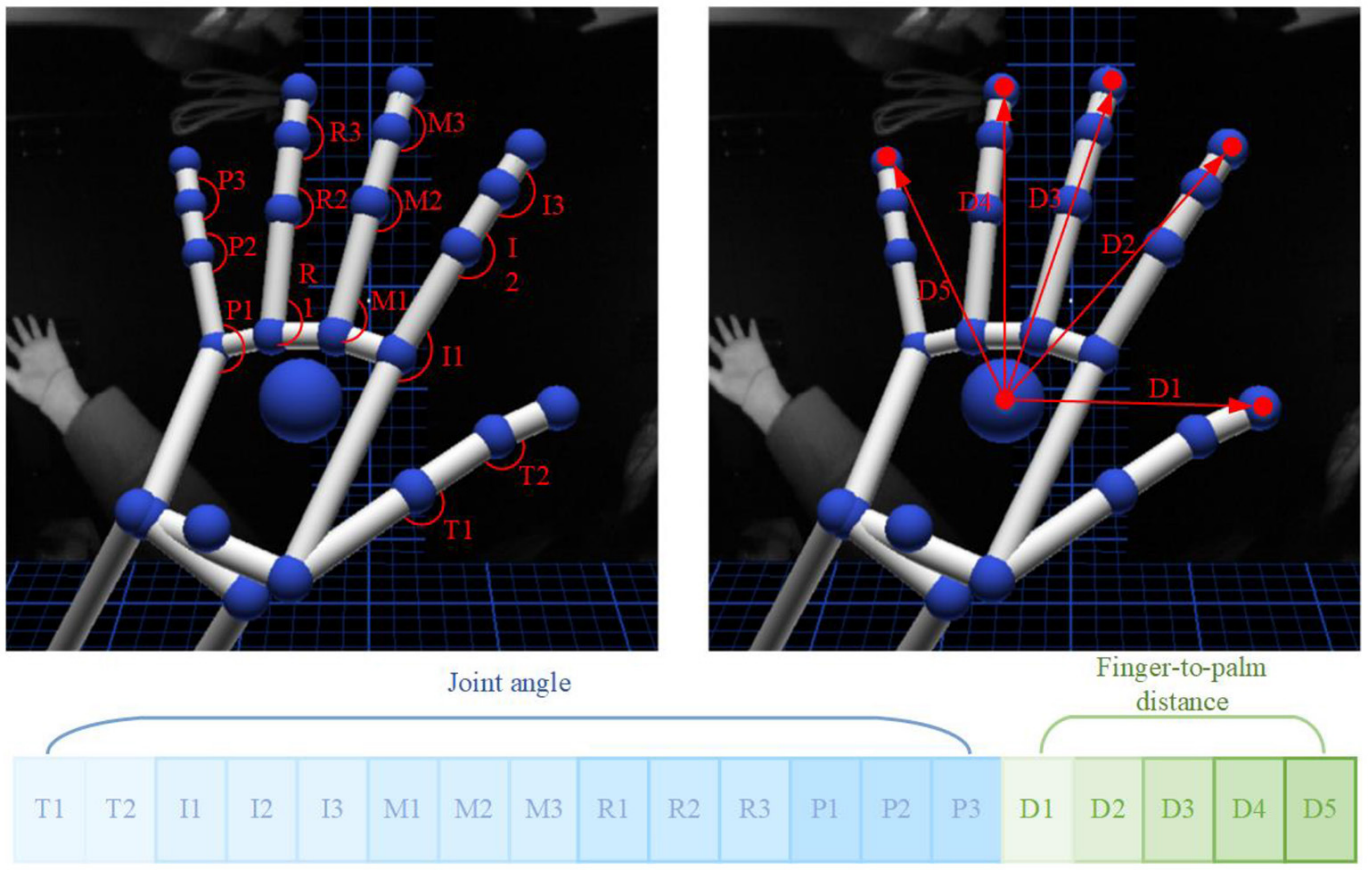
Joint angle and finger-to-palm distance kinematic features were extracted from Leap motion controller.

Similar to EMG processing, Leap motion signal of middle 3s were used in the analysis. A 250-ms-lengths sliding window with an overlap of 50 ms was used to segment leap motion data. For each window, the average of the five frames is extracted as the kinematics data. Each frame of leap motion controller contains instant hand skeleton data of the recorded scene. In the experiment, Leap motion controller was recording at 20 frames per second. Extracted joint angle and finger-to-palm distance were used as kinematic features for recognition of different patterns. A 19-dimensional feature sequence is then established in each frame of kinematic data, see Figure 3. Among them, T1 and T2 are the joint angles of the thumb, I1–I3 are the joint angles of index finger, M1–M3 are the joint angles of middle finger, R1–R3 are the joint angles of ring finger, P1–P3 are joint angles of the little thumb, and distances from each of the five fingertip to palm are given as D1–D5.

1) Joint Angle

The joint angle refers to the angle between the joints of the palm, that is, the angle formed between Metacarpal and Proximal, Proximal and Intermediate, and Intermediate and Distal of each finger. The calculation formula is:

$$\begin{array}{l} \theta =\arccos\frac{\overset{\to }{u}\cdot \overset{\to }{v}}{|\overset{\to }{u}|\cdot |\overset{\to }{v|}} \end{array}$$

$\vec{u}$ and $\vec{v}$ are the two adjacent parts linked to the joint of a finger. The calculated joint angle range is [0°,180°]. When the palm is fully opened, the angle value of each joint reaches the maximum. Since the Thumb finger does not have Metacarpal, there are only two joint angles, and the remaining fingers have three joint angles each. Thus, a total of 14 joint angle feature values were used for one hand, see Figure 3.

2) Finger-to-Palm Distance

The distance from the fingertip to the palm is calculated as the Euclidean distance between the fingertip of each finger and the palm. The calculation formula is:

$$\begin{array}{l} D_{i}=||{\text{P}}_{\text{i}}-O|{|}_{,i=1,2,3,4,5} \end{array}$$

Point Pi is the position coordinate of the fingertip of each finger, and point O is the position coordinate of the palm. When the palm is fully opened the distance from the fingertip to the palm reaches the maximum. A total of five fingertip distance were used as distances features, see Figure 3.

### Proposed Classification Framework

**Figure 4:**
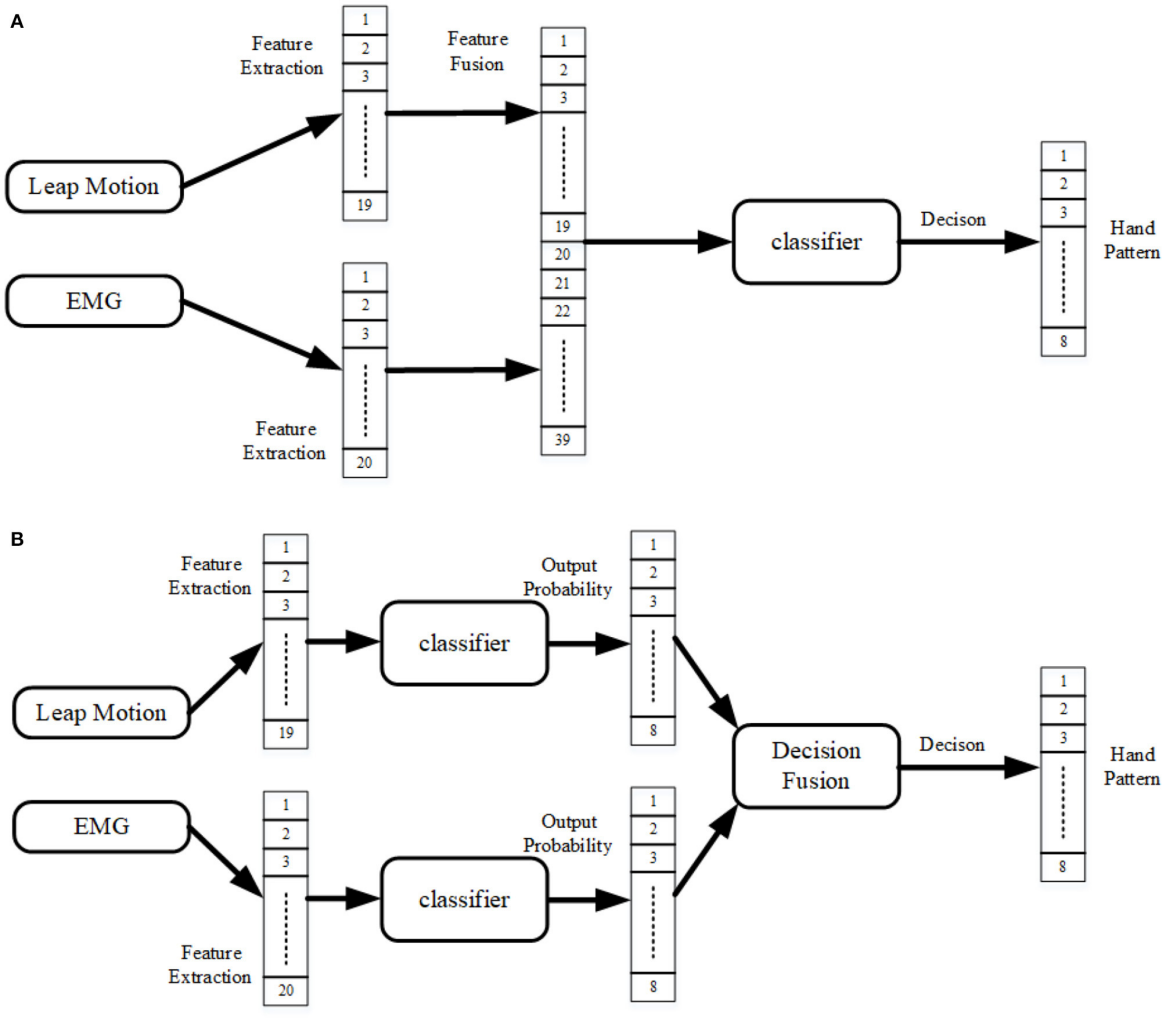
Proposed classification framework of the system. **(A)** Feature fusion of EMG and kinematics (leap motion); **(B)** Decision fusion of EMG and kinematics (leap motion).

Feature fusion and decision fusion of EMG and kinematics were used to achieve hand pattern recognition, respectively. Among them, the feature sequences extracted from EMG data and Leap Motion data were fused together as inputs to the classifier, which is called feature fusion shown in Figure 4A. For decision fusion, the Leap Motion feature and the EMG feature were first input to the classifier separately, then the probabilities output of the two classifiers were superimposed to finally obtain the classification result, see Figure 4B.

Linear discrimination analysis (LDA) is an easy and effective classifier that was used because it gives high performance despite low computational cost and robustness for long term usage (Chen et al., 2013). The experiment uses a 10-fold cross-validation method to train the classifier multiple times to ensure that each data can be used as training data and test data. The accuracy of each test is the ratio of the number of correctly recognized patterns to the total number of tested patterns. The classification rate of each subject is taken as the average of 10 times of cross-validation training results. In this experiment, the final accuracies were calculated as the mean of 10 healthy subjects and five post stroke patients. Python programming language was used to implement the classification algorithm. One-Way ANOVA was used to compare the classification results of EMG, kinematics, feature fusion and decision fusion of EMG and kinematics, respectively. The significance threshold was set to 0.05 in the experiment.

### Noise Contamination

Performance of a classification model in the presence of noise is an important area to address. Hence, we must assess the model's tolerance of input perturbations. For that reason, three different levels of Gaussian noise are added to the raw EMG data to contaminate it and then the contaminated data was fed to the classifiers. Since raw inputs' given order of magnitude is 10^−5^, we set the noise levels accordingly to 1 × 10^−5^, 2 × 10^−5^, and 1 × 10^−4^ (Jia, 2020).

## Result

### Comparison of Classification Performance for Different Fusion Strategy

**Figure 5:**
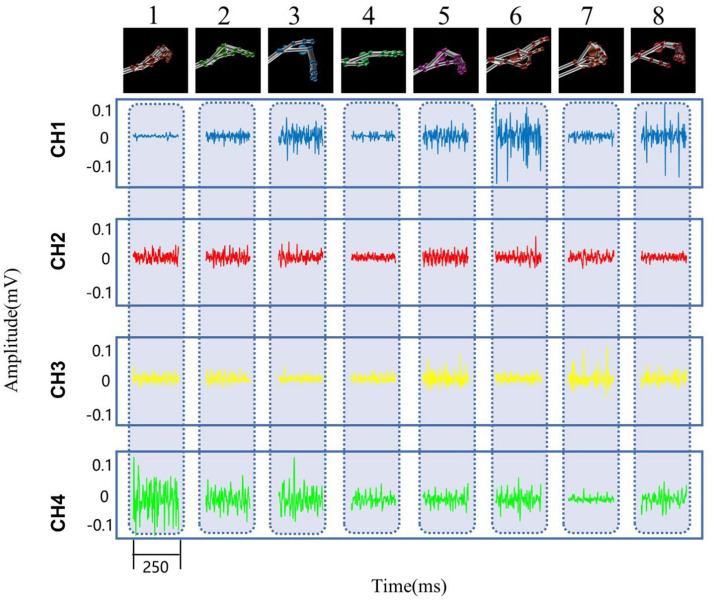
Representative EMG and kinematic data of eight hand patterns recorded from one subject. Patterns 1–8 are: finger bend, finger close, finger flexion, hand open, lateral pinch, fingertip to fingertip, fist, and cylindrical grip, respectively. The top row kinematic data are recorded from Leap motion. The EMG data of CH1–CH4 are recorded from muscles of abductor pollicis brevis, flexor carpi radialis, extensor digitorum, and extensor carpi ulnaris, respectively.

Representative EMG and kinematic data of different patterns from one normal subject are shown in Figure 5. In the figure, EMG signals are recorded from muscles of abductor pollicis brevis, flexor carpi radialis, extensor carpi ulnaris, and extensor digitorum. It can be observed that muscle activity patterns are different within four muscles during different tasks. In addition, Leap motion sensor were used to track the participant's hand to monitor the kinematics. Then the provided features of joint angles and finger-to-palm distances were estimated from the kinematics data. It can be seen from the figure that the kinematics are quite different between the eight hand patterns.

**Figure 6:**
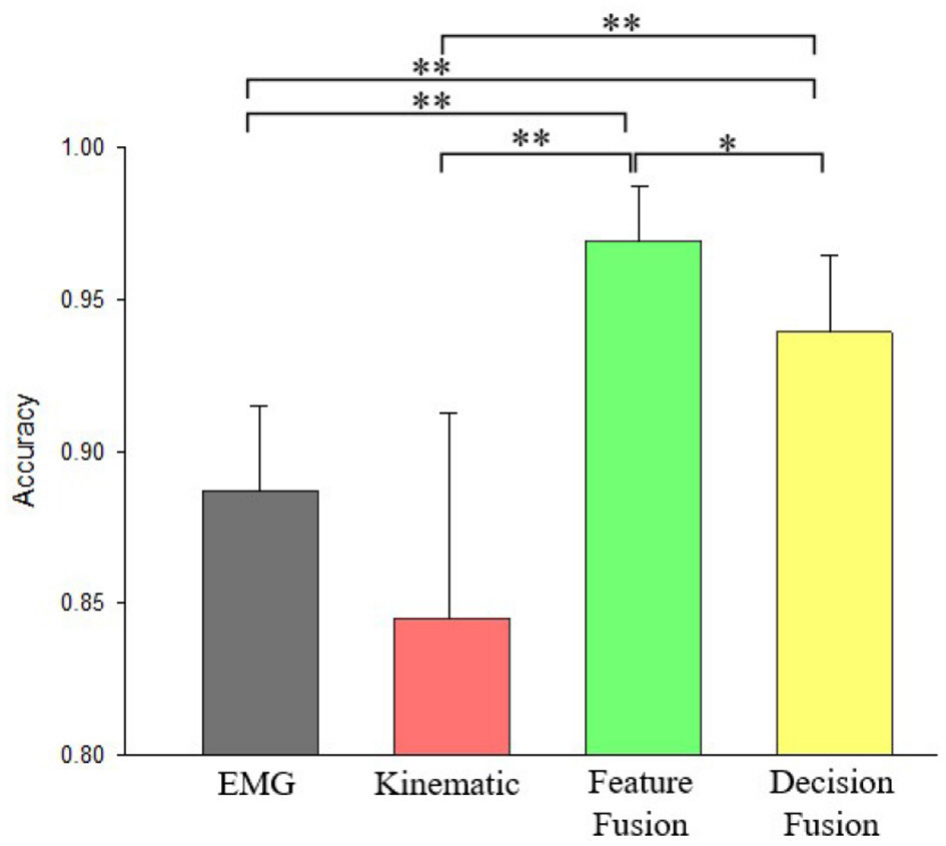
Accuracy comparisons of pattern recognition using EMG only, kinematics only, feature fusion of kinematics+EMG, and decision fusion of kinematics+EMG for normal subjects. * Denotes *p* < 0.05, ** Denotes *p* < 0.01.

For 10 normal subjects, average hand pattern recognition accuracy was 88.71 ± 2.79% for EMG features only, 84.49 ± 6.77% for kinematic features only, 96.90 ± 1.81% for feature fusion of EMG and kinematics, 93.91 ± 2.57 % for decision fusion of EMG and kinematics (Figure 6). It can be observed after feature fusion that classification accuracy have increased to 96.90% and standard deviation have decreased to 1.81%. From the Figure 6, it can also be seen that the accuracy of pattern recognition using feature fusion of EMG and kinematic is significantly larger than that of using kinematic features alone (*p* = 0.001). Besides, decision fusion method comparison with kinematics only was statistically highly significant as well (*p* = 0.01). Moreover, there was significant difference between EMG and decision fusion of EMG and kinematic (*p* = 0.003,). In addition, there was no significant difference between kinematic features only and EMG features only in classification rate. Difference between feature fusion and decision fusion of kinematics and EMG was significant (*p* = 0.05).

**Figure 7:**
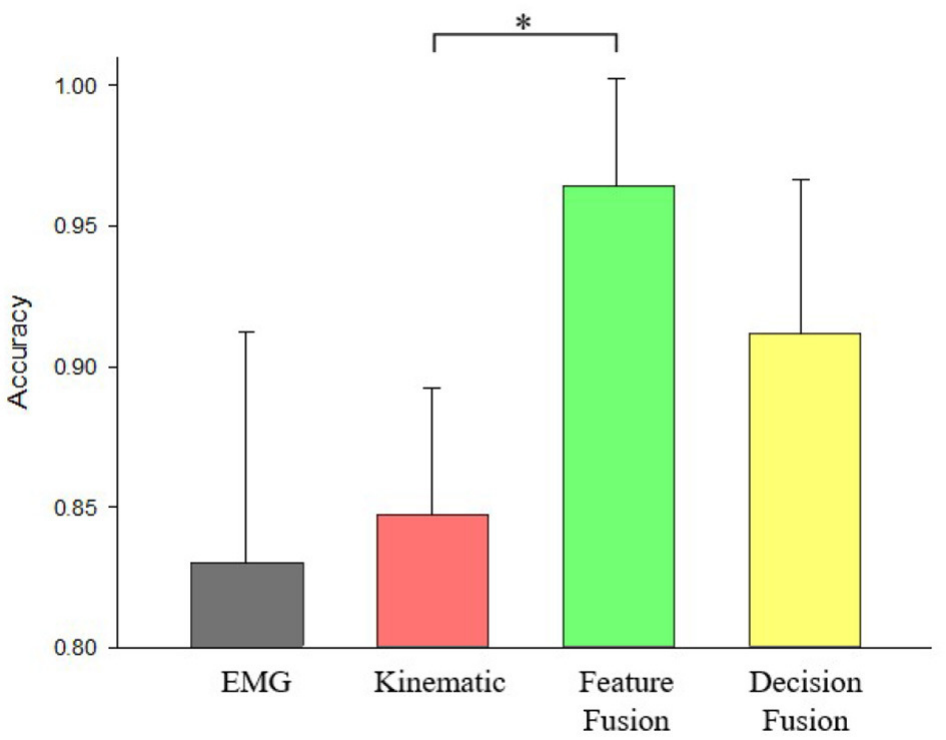
Accuracy comparisons of pattern recognition using EMG only, kinematics only, feature fusion of kinematics+EMG, and decision fusion of kinematics+EMG for post stroke patients. * Denotes *p* < 0.05.

For the post stroke patients, hand pattern recognition accuracy was 83 ± 8.21% for EMG features only, 84.71 ± 4.54% for kinematic features only, 96.43 ± 3.83% for feature fusion of EMG and kinematics, 91.18 ± 5.50% for decision fusion of EMG and kinematics (Figure 7). It is observable that both fusion methods are able to achieve average accuracy higher than 90%. From Figure 7, it is noticeable that pattern recognition accuracy using fusion features of kinematics and EMG is significantly larger than solely using kinematics features (*p* = 0.019). Additionally, there was no significant difference in classification accuracy between decision fusion of kinematics and EMG method and kinematics features only method (*p* = 0.151).

### Classification Performance With Noise on EMG Recordings

Comprehensive comparison between three levels of noisy contaminated data was made for EMG only, decision fusion and feature fusion of EMG and kinematics. Table 2 displays robustness result for normal subjects and post stroke subjects. It can be observed from Table 2 that classifiers realize near exact test accuracy when provided with 1 × 10^−5^ level of additive noise. But 1 × 10^−4^ level of additive noise decreases test accuracy greatly for EMG only classifier for both normal subjects and post stroke subjects. For 1 × 10^−4^ level of noisy data EMG decreases test accuracy from 85.91 to 58.88% for normal subjects and from 81.07 to 53.50% for post stroke patients. But test accuracy of decision fusion and feature fusion did not suffer such sharp decrease. For decision fusion decrease was from 93.54 to 88.13% for normal subjects and from 91.11 to 86.04% for post stroke patients. Similarly, feature fusion decrease was from 96.61 to 90.13% for normal subjects and from 95.61 to 91.07% for post stroke patients. It is distinguished that feature fusion method provides best performance which is robust to noises.

**Table 2:** Test accuracy of classifiers when given different levels of noise.

**Noise level**	**Normal subjects**			**Patient after stroke**		
	**EMG**	**Feature fusion**	**Decision fusion**	**EMG**	**Feature fusion**	**Decision fusion**
1e−5	85.91%	93.54%	96.61%	81.07%	91.11%	95.61%
2e−5	81.87%	92.37%	95.57%	77.21%	89.89%	95.39%
1e−4	58.88%	88.13%	90.13%	53.50%	86.04%	91.07%

### Classification Performance With Different Channel Combinations

Comparison of hand pattern recognition accuracy between different classification models are made with 15 different channels combinations. Combinations are as following: four single channels, six dual channels, four three channel combinations, and one with all four channels combined. Investigation is carried out for both normal subjects and post stroke patients.

**Figure 8:**
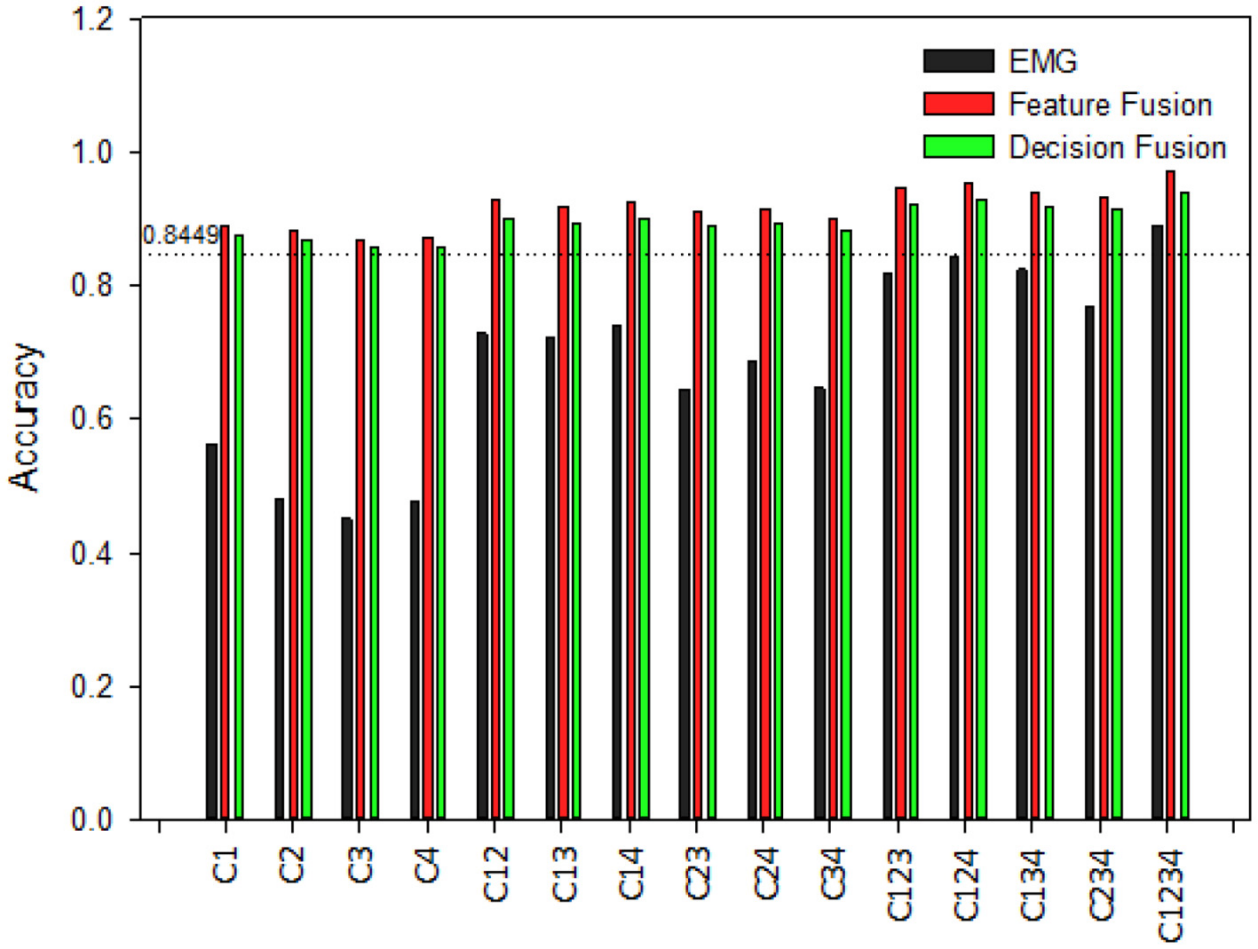
Classification accuracy of different channel combinations for hand pattern recognition for normal subjects. C1–C4 are recorded from muscles of abductor pollicis brevis, flexor carpi radialis, extensor digitorum and extensor carpi ulnaris, respectively.

Figure 8 displays recognition accuracy with different channels combination for normal subjects. The line showing 0.8449 represents pattern recognition accuracy using kinematics only. By increasing EMG channels from one to four, the overall classification accuracies are increased for EMG only classifier. When a single EMG channel is used for recognition, the highest recognition accuracy is produced by C1 for EMG only classifier. For dual channel combinations, C23 produced lowest accuracy, and C14 produced highest accuracy for EMG only classifier. Furthermore, between three channel combinations, C124 and C234 produced highest and lowest recognition accuracy for EMG only classifiers. And from the Figure 8 we can see combining all four channels into one yields the best overall accuracy for all classifiers. Even with cases of single EMG channel, it is evident the test accuracy is still high after fusion with kinematics. This fusion based method makes the classifier robust despite potential failure of specific EMG channels. Furthermore, feature fusion method produces slightly better classification performance than decision fusion method in this study.

**Figure 9:**
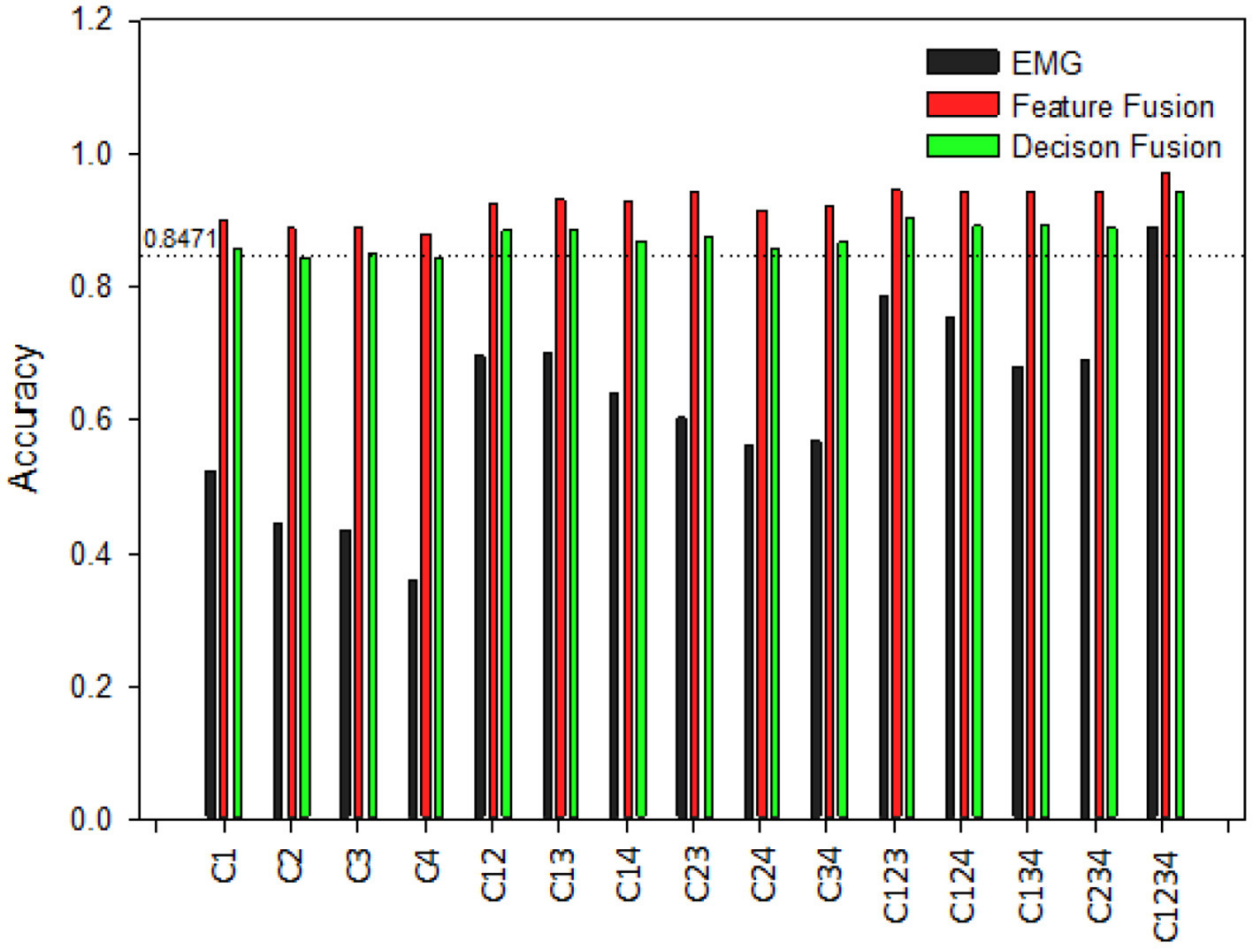
Classification accuracy of different channel combinations for hand pattern recognition for post stroke patients. C1–C4 are recorded from muscles of abductor pollicis brevis, flexor carpi radialis, extensor digitorum, and extensor carpi ulnaris, respectively.

On the other hand, Figure 9 shows the recognition results with different channels for post stroke patients. From Figure 9 it is visible that EMG only classifier produces lower test accuracy for post stroke patients than normal subjects. Classification accuracies are increased by rising the number of EMG channels, just as observed for normal subjects. Besides, it was apparent for both normal subjects and post stroke patients that channel combinations that included the channel C1 gave better performance than the combinations that didn't include this specific channel. This suggests C1 would be the optimal recording site for hand pattern recognition with EMG based classifier. Moreover, the lower classification accuracy were improved by fusion with kinematics. After fusion of EMG data with kinematics the classification performances among post stroke patient group are very close to results obtained for normal subjects.

## Discussion

In this study, a pattern recognition method was proposed that combined EMG with kinematics data for classification toward application in upper limb assistive and rehabilitation robotics. The feasibility of the proposed method was demonstrated by conducting experiments on 10 normal subjects and five post stroke patients. Classification accuracy was improved with the fusion method as only EMG and kinematics classification separately were unsatisfactory. Robustness of the model was validated with noise and channel combination comparison.

### Fusion of EMG With Kinematics for Hand Pattern Recognition

EMG has been widely used for pattern recognition for many years, especially for applications in prosthesis (Lee and Saridis, 1984; Ajiboye and Weir, 2005; Kuiken et al., 2009). The use of EMG in driving assistive and rehabilitation robotics post stroke is increasing recently (Hu et al., 2013; Leonardis et al., 2015; Park et al., 2020). However, the EMG based pattern recognition performance in assistive and rehabilitation robotics post stroke remains unsatisfactory (Lu et al., 2019). In recent years, kinematic sensors have been increasingly used for hand pattern recognition. Various types of kinematics sensors were used for pattern recognition, such as motion capture devices (Pun et al., 2011), inertial measurement unit (IMU) (Kim et al., 2019), and strain sensors (Ferrone et al., 2016) etc. However, most commercially available kinematics devices are expensive and complex to set up such as wearable data gloves, Vicon motion sensor system. While on the other hand, leap motion device is a cheap, convenient and markerless hand kinematic recording device. Hence, combining kinematics obtained from leap motion device with EMG was interesting to make the proposed model cost-effective.

In this study, we propose a method of combining EMG and kinematics together for hand pattern recognition toward assistive and rehabilitation robots. The proposed fusion methods of EMG and kinematics can improve classification accuracy of hand patterns in daily activities. Experiments using proposed model of feature fusion and decision fusion was conducted on 10 normal subjects and five post stroke patients showed increased classification accuracy. This classification accuracy is comparable to other sensors fusion method such as EMG and force myograph (FMG) (Jiang et al., 2020), EMG and IMU (Georgi et al., 2015), and EMG and strain sensors (Landgraf et al., 2018). However, Leap motion is easy to use and off-the-shelf compared with these sensors.

To our great knowledge, very few works has been reported about the fusion of EMG and Leap motion based kinematics for hand pattern recognition of daily activities. High accuracy was successfully achieved for post stroke patients by implementing the proposed method of feature fusion and decision fusion from EMG channels and Leap motion. The model obtained satisfactory test accuracy of 96.43% for feature fusion and 91.18% for decision fusion of EMG and kinematics. Ricardez et al. (2018) conducted similar experiment on only three normal subjects using 8-channel EMG and leap motion but overall classification accuracy was <90%. No publication yet have provided experimental results on stroke patients with fusion of EMG and Leap motion for hand pattern recognition. Highly accurate, cost-effective, and limited EMG electrode nature of the fusion method would be meaningful in a practical sense.

### Robustness Analysis of the Proposed Methods

Checking robustness of the proposed method is imperative to investigate its stability against different levels of noise. Table 2 displayed comparison of classification models after fed with three different level of noise. The chosen levels of noise were 1 × 10^−5^, 2 × 10^−5^, and 1 × 10^−4^. Table 2 showed great decrease in test accuracy for EMG only classifier whereas, the proposed feature fusion and decision fusion of EMG and kinematics classifiers saw small decrease meaning they are robust to EMG noise.

To check robustness of the classifier on the condition that one or multiple EMG channels fail, we compare the classification performance of 15 different EMG channel combinations. If channels fails, the overall classification accuracies would decrease with the number of channels. However, after fusion with kinematics the classifier would be robust from channels failure. It was observed that after fusion of single channel EMG with kinematics, the classification accuracy could reach close to 90% for both normal subjects and post stroke patients. Hence, the proposed fusion method would use minimal number of EMG electrodes without the deterioration of the classification accuracy. To our great knowledge, very few publications have verified robustness of fusion of EMG and Leap motion.

### Limitation of the Study

In this study, FCA and FCA-C score shows that the five recruited patients had mild to no restrictions on activities of daily life. However, most post stroke patients have low FCA score and require assistance of robotics for daily activities. Lu et al. (2019) reported the feasibility of hand pattern recognition under the assistance of robotic hand on post stroke patients with FCA-C score ranging from 0 to 7. In this study, rehabilitation and assistive robotics haven't yet been implemented for stroke patients. In future, we would test our classification accuracy on stroke patients with lower FCA score with the help of assistive and rehabilitation robotics.

The proposed method uses simple feature fusion and decision fusion because the main purpose of this study was to validate the feasibility of fusion with EMG and kinematics for hand pattern recognition in normal subjects and stroke patients. The results produced an average accuracy of above 90% for both feature fusion and decision fusion method. In the future, we would use more complex algorithms to optimize fusion methods and further improve the classification accuracy. Another limitation of the proposed method is that the dataset were trained offline and no online experiment was done. Future work will focus on real-time pattern recognition using the method presented here.

## Conclusion

This paper proposes a method that applies feature fusion and decision fusion using EMG features and kinematic features for hand pattern recognition toward application in upper limb assistive and rehabilitation robotics. The results showed that the infused kinematic features could improve the classification accuracy compared with EMG features only for hand pattern recognition of ADLs. Robustness of the proposed method was demonstrated by adding noise to EMG data and comparison between different channel combinations. Classification accuracy of feature fusion and decision fusion was above 90% for both normal subjects and post stroke patients which means that the system has significant potential in the field of assistive and rehabilitation robotics. Future work will be conducted with real-time pattern classification on stroke survivors.

## Data Availability Statement

The raw data supporting the conclusions of this article will be made available by the authors, without undue reservation.

## Ethics Statement

The studies involving human participants were reviewed and approved by Nanjing Brain Hospital Affiliated to Nanjing Medical University. The patients/participants provided their written informed consent to participate in this study.

## Author Contributions

HZ and XZ initiated and supervised the research project. QZ, MZ, JR, SW, and LZ carried out the research. HZ, XZ, QZ, and SS wrote part of the manuscript. QZ, MZ, XZ, SW, LZ, JR, and HZ analyzed the results and prepared the figures and tables. All the authors contributed to the article and approved the submitted version.

## Conflict of Interest

The authors declare that the research was conducted in the absence of any commercial or financial relationships that could be construed as a potential conflict of interest.

## References

[B1] Ajiboye A. B. Weir R. F. (2005). A heuristic fuzzy logic approach to EMG pattern recognition for multifunctional prosthesis control. IEEE Trans. Neural Syst. Rehabil. Eng. 13, 280–291. 10.1109/TNSRE.2005.847357 16200752

[B2] Arteaga M. V. Castiblanco J. C. Mondragon I. F. Colorado J. D. Alvarado-Rojas C. (2020). EMG-based adaptive trajectory generation for an exoskeleton model during hand rehabilitation exercises, in 2020 8th IEEE RAS/EMBS International Conference for Biomedical Robotics and Biomechatronics (BioRob). Presented at the 2020 8th IEEE RAS/EMBS International Conference for Biomedical Robotics and Biomechatronics (BioRob) (New York, NY: IEEE), 416–421.

[B3] Bachmann D. Weichert F. Rinkenauer G. (2018). Review of three-dimensional human-computer interaction with focus on the leap motion controller. Sensors 18:2194. 10.3390/s18072194 29986517 PMC6068627

[B4] Bernhardt J. Mehrholz J. (2019). Robotic-assisted training after stroke: RATULS advances science. Lancet 394, 6–8. 10.1016/S0140-6736(19)31156-0 31128923

[B5] Castiblanco J. C. Ortmann S. Mondragon I. F. Alvarado-Rojas C. Jöbges M. Colorado J. D. (2020). Myoelectric pattern recognition of hand motions for stroke rehabilitation. Biomed. Signal Process. Control 57:101737. 10.1016/j.bspc.2019.101737

[B6] Chen M. Cheng L. Huang F. Yan Y. Hou Z.-G. (2017). Towards robot-assisted post-stroke hand rehabilitation: fugl-meyer gesture recognition using sEMG, in 2017 IEEE 7th Annual International Conference on CYBER Technology in Automation, Control, and Intelligent Systems (CYBER). Presented at the 2017 IEEE 7th Annual International Conference on CYBER Technology in Automation, Control, and Intelligent Systems (CYBER) (Honolulu, HI: IEEE), 1472–1477.

[B7] Chen X. Zhang D. Zhu X. (2013). Application of a self-enhancing classification method to electromyography pattern recognition for multifunctional prosthesis control. J. NeuroEng. Rehabil. 10:44. 10.1186/1743-0003-10-44 23634939 PMC3689085

[B8] Cohen M. W. (2020). Hand rehabilitation assessment system using leap motion controller. AI Soc. 35, 581–594. 10.1007/s00146-019-00925-8

[B9] Dipietro L. Ferraro M. Palazzolo J. J. Krebs H. I. Volpe B. T. Hogan N. (2005). Customized interactive robotic treatment for stroke: EMG-triggered therapy. IEEE Trans. Neural Syst. Rehabil. Eng. 13, 325–334. 10.1109/TNSRE.2005.850423 16200756 PMC2752646

[B10] Englehart K. Hudgins B. (2003). A robust, real-time control scheme for multifunction myoelectric control. IEEE Trans. Biomed. Eng. 50, 848–854. 10.1109/TBME.2003.813539 12848352

[B11] Feigin V. L. Forouzanfar M. H. Krishnamurthi R. Mensah G. A. Connor M. Bennett D. A.. (2014). Global and regional burden of stroke during 1990–2010: findings from the Global Burden of Disease Study 2010. Lancet 383, 245–255. 10.1016/S0140-6736(13)61953-4 24449944 PMC4181600

[B12] Ferrone A. Maita F. Maiolo L. Arquilla M. Castiello A. Pecora A.. (2016). Wearable band for hand gesture recognition based on strain sensors, in 2016 6th IEEE International Conference on Biomedical Robotics and Biomechatronics (BioRob). Presented at the 2016 6th IEEE International Conference on Biomedical Robotics and Biomechatronics (BioRob) (Singapore: IEEE), 1319–1322.

[B13] Geng Y. Zhang L. Tang D. Zhang X. Li G. (2013). Pattern recognition based forearm motion classification for patients with chronic hemiparesis, in 2013 35th Annual International Conference of the IEEE Engineering in Medicine and Biology Society (EMBC). Presented at the 2013 35th Annual International Conference of the IEEE Engineering in Medicine and Biology Society (EMBC) (Osaka: IEEE), 5918–5921. 10.1109/EMBC.2013.6610899 24111086

[B14] Georgi M. Amma C. Schultz T. (2015). Recognizing hand and finger gestures with IMU based motion and EMG based muscle activity sensing, in Proceedings of the International Conference on Bio-Inspired Systems and Signal Processing. Presented at the International Conference on Bio-Inspired Systems and Signal Processing (Lisbon: SCITEPRESS–Science and and Technology Publications), 99–108.

[B15] He J. Zhang D. Jiang N. Sheng X. Farina D. Zhu X. (2015). User adaptation in long-term, open-loop myoelectric training: implications for EMG pattern recognition in prosthesis control. J. Neural Eng. 12:046005. 10.1088/1741-2560/12/4/046005 26028132

[B16] Hu X. L. Tong K. Y. Wei X. J. Rong W. Susanto E. A. Ho S. K. (2013). T he effects of post-stroke upper-limb training with an electromyography (EMG)-driven hand robot. J. Electromyogr. Kinesiol. 23:1065–1074. 10.1016/j.jelekin.2013.07.007 23932795

[B17] Jia G. (2020). Classification of electromyographic hand gesture signals using modified fuzzy C-means clustering and two-step machine learning approach. IEEE Trans. Neural Syst. Rehabil. Eng. 28:8. 10.1109/TNSRE.2020.2986884 32286995

[B18] Jiang S. Gao Q. Liu H. Shull P. B. (2020). A novel, co-located EMG-FMG-sensing wearable armband for hand gesture recognition. Sens. Actuators Phys. 301:111738. 10.1016/j.sna.2019.111738

[B19] Kim M. Cho J. Lee S. Jung Y. (2019). IMU sensor-based hand gesture recognition for human-machine interfaces. Sensors 19:3827. 10.3390/s19183827 31487894 PMC6767360

[B20] Kuiken T. A. Li G. Lock B. A. Lipschutz R. D. Miller L. A. Stubblefield K. A.. (2009). Targeted muscle reinnervation for real-time myoelectric control of multifunction artificial arms. JAMA. 301, 619–628. 10.1001/jama.2009.116 19211469 PMC3036162

[B21] Landgraf M. Yoo I. S. Sessner J. Mooser M. Kaufmann D. Mattejat D.. (2018). Gesture recognition with sensor data fusion of two complementary sensing methods^*^, in 2018 7th IEEE International Conference on Biomedical Robotics and Biomechatronics (Biorob). Presented at the 2018 7th IEEE International Conference on Biomedical Robotics and Biomechatronics (Biorob) (Enschede: IEEE), 795–800.

[B22] Lee S. Saridis G. (1984). The control of a prosthetic arm by EMG pattern recognition. IEEE Trans. Automat. Control 29, 290–302. 10.1109/TAC.1984.11035219865887

[B23] Lee S. W. Wilson K. M. Lock B. A. Kamper D. G. (2011). Subject-specific myoelectric pattern classification of functional hand movements for stroke survivors. IEEE Trans. Neural Syst. Rehabil. Eng. 19:9. 10.1109/TNSRE.2010.2079334 20876030 PMC4010155

[B24] Lenzi T. De Rossi S. M. M. Vitiello N. Carrozza M. C. (2011). Proportional EMG control for upper-limb powered exoskeletons, in 2011 Annual International Conference of the IEEE Engineering in Medicine and Biology Society. Presented at the 2011 33rd Annual International Conference of the IEEE Engineering in Medicine and Biology Society (Boston, MA: IEEE), 628–631. 10.1109/IEMBS.2011.6090139 22254387

[B25] Leonardis D. Barsotti M. Loconsole C. Solazzi M. Troncossi M. Mazzotti C. (2015) An EMG-controlled robotic hand exoskeleton for bilateral rehabilitation. IEEE Trans. Haptics. 8, 140–151. 10.1109/TOH.2015.2417570. 25838528

[B26] Li H. Wu L. Wang H. Han C. Quan W. Zhao J. (2020). Hand gesture recognition enhancement based on spatial fuzzy matching in leap motion. IEEE Trans. Ind. Inform. 16, 1885–1894. 10.1109/TII.2019.2931140

[B27] Lu W. Tong Z. Chu J. (2016). Dynamic hand gesture recognition with leap motion controller. IEEE Signal Process. Lett. 23, 1188–1192. 10.1109/LSP.2016.259047032276493

[B28] Lu Z. Tong K. Zhang X. Li S. Zhou P. (2019). Myoelectric pattern recognition for controlling a robotic hand: a feasibility study in stroke. IEEE Trans. Biomed. Eng. 66:8. 10.1109/TBME.2018.2840848 29993410

[B29] Mantecón T. del-Blanco C. R. Jaureguizar F. García N. (2019). A real-time gesture recognition system using near-infrared imagery. PLoS ONE 14:e0223320. 10.1371/journal.pone.0223320 31581266 PMC6776328

[B30] Mehrholz J. Pohl M. Platz T. Kugler J. Elsner B. (2018). Electromechanical and robot-assisted arm training for improving activities of daily living, arm function, and arm muscle strength after stroke. Cochrane Database Syst. Rev. 2015:CD006876. 10.1002/14651858.CD006876.pub5 26559225 PMC6465047

[B31] Nizamis K. Rijken N. Mendes A. Janssen M. Bergsma A. Koopman B. (2018). A novel setup and protocol to measure the range of motion of the wrist and the hand. Sensors 18:3230. 10.3390/s18103230 30257521 PMC6210232

[B32] Nogales R. Benalcazar M. (2019). Real-time hand gesture recognition using the leap motion controller and machine learning, in 2019 IEEE Latin American Conference on Computational Intelligence (LA-CCI). Presented at the 2019 IEEE Latin American Conference on Computational Intelligence (LA-CCI) (Guayaquil: IEEE), 1–7.

[B33] Park S. Fraser M. Weber L.M. Meeker C. Bishop L. Geller D.. (2020). User-driven functional movement training with a wearable hand robot after stroke. IEEE Trans. Neural Syst. Rehabil. Eng. 28, 2265–2275. 10.1109/TNSRE.2020.3021691 32886611 PMC8274060

[B34] Pun C.-M. Zhu H.-M. Feng W. (2011). Real-time hand gesture recognition using motion tracking. Int. J. Comput. Intell. Syst. 4, 277–286. 10.1080/18756891.2011.9727783

[B35] Ricardez G. A. G. Ito A. Ding M. Yoshikawa M. Takamatsu J. Matsumoto Y.. (2018). Wearable device to record hand motions based on EMG and visual information, in 2018 14th IEEE/ASME International Conference on Mechatronic and Embedded Systems and Applications (MESA). Presented at the 2018 14th IEEE/ASME International Conference on Mechatronic and Embedded Systems and Applications (MESA) (Oulu: IEEE),1–6.

[B36] Rose C. G. Pezent E. Kann C. K. Deshpande A. D. O'Malley M. K. (2018). Assessing wrist movement with robotic devices. IEEE Trans. Neural Syst. Rehabil. Eng. 26, 1585–1595. 10.1109/TNSRE.2018.2853143 29994401

[B37] Song R. Tong K. Hu X. Zhou W. (2013). Myoelectrically controlled wrist robot for stroke rehabilitation. J. NeuroEng. Rehabil. 10:52. 10.1186/1743-0003-10-52 23758925 PMC3685570

[B38] Vermillion B. C. Dromerick A. W. Lee S. W. (2019). Toward restoration of normal mechanics of functional hand tasks post-stroke: subject-specific approach to reinforce impaired muscle function. IEEE Trans. Neural Syst. Rehabil. Eng. 27, 1606–1616. 10.1109/TNSRE.2019.2924208 31226079 PMC6713235

